# Overnight deep inspiration patterns in obstructive sleep apnea patients with and without asthma

**DOI:** 10.14814/phy2.70622

**Published:** 2025-11-02

**Authors:** Shokoufeh Mousavi, Maryam Mohebbi, Parisa Adimi Naghan, Azadeh Yadollahi

**Affiliations:** ^1^ Department of Biomedical Engineering, Faculty of Electrical Engineering K. N. Toosi University of Technology Tehran Iran; ^2^ Chronic Respiratory Diseases Research Center, National Research Institute of Tuberculosis and Lung Diseases Shahid Beheshti University of Medical Sciences Tehran Iran; ^3^ Institute of Biomedical Engineering University of Toronto Toronto Ontario Canada; ^4^ KITE, Toronto Rehabilitation Institute University Health Network Toronto Ontario Canada

**Keywords:** asthma, bronchodilation, deep breathing, modeling, obstructive sleep apnea

## Abstract

Asthma and obstructive sleep apnea (OSA) often co‐occur, exacerbating respiratory difficulties and altering airway physiology during sleep. Respiratory obstructions in OSA usually terminate with deep inspiration, and in asthma, deep inspiration may function as a bronchodilator or induce bronchoconstriction. This study investigated deep inspiration patterns using polysomnography data from 202 OSA patients (Apnea‐Hypopnea Index (AHI) > 5) in the Sleep Heart Health Study, including asthma patients and matched controls. Airflow signals were used to calculate the average amplitude of three post‐event deep breaths (PEDB), as overshoots usually peak within these breaths. PEDB curves were compared within and between groups, each including 68 mild, 19 moderate, and 14 severe OSA cases (AHI > 30). In severe OSA, mean PEDB increased from the first to last sleep quartile in controls (*p* < 0.05) but showed no change in those with asthma (*p* > 0.05). PEDB values were higher in controls than in asthma patients with moderate and severe OSA. Reduced PEDB intensity in severe OSA with asthma suggests impaired bronchodilator effects of deep inspirations, possibly from chronic inflammation and fluid shifts. These findings enhance understanding of asthma–OSA interactions and the potential role of deep inspirations in mitigating overnight narrowing in lower airways.

## 1 INTRODUCTION

Obstructive sleep apnea (OSA) and asthma are two highly prevalent chronic conditions in adults that frequently coexist (Pardo‐Manrique et al., 2024). Asthma affects over 200 million people globally and OSA prevalence is 19%–60% in patients with asthma (Davies et al., 2019; Kong et al., 2017; Prasad et al., 2020). Asthma is marked by constriction of the lower airways leading to shortness of breath, wheezing, and disabilities (Mauer & Taliercio, 2020). OSA is a common risk factor for nocturnal worsening of asthma symptoms and a sign of poor asthma control (Kong et al., 2017). Asthma and OSA share common risk factors, such as obesity, age, and inflammation, which may contribute to their co‐existence. However, recent studies suggest that asthma–OSA overlap may have synergistic effects, with each condition potentially intensifying the severity of the other rather than simply coexisting (He et al., 2019; Saxena et al., 2023).

OSA is characterized by repeated episodes of airflow cessation (apnea) or reduction (hypopnea) during sleep, due to the collapse of the pharyngeal airway (Gottlieb & Punjabi, 2020). Several physiological mechanisms have been proposed to explain how OSA worsens asthma, including hypoxemia, circadian changes in autonomic function, and reduced lung volume (Brockmann et al., 2014; Castro‐Rodriguez et al., 2017; Prasad et al., 2014; Prasad et al., 2020). Also, the chronic low‐grade inflammation is present in both asthma and OSA, and both conditions lead to upregulation of similar inflammatory pathways which may contribute to the synergistic interactions between asthma and OSA (He et al., 2019; Sánchez et al., 2016). Moreover, recent studies have highlighted the interconnection between the pharyngeal and lower airways as the “unified airway”, which hypothesizes that inflammation or obstruction in one segment of the airway can influence the other segment (Ahookhosh et al., 2020). Studies have shown that conditions like rhinitis, which affect the upper airway, can exacerbate asthma symptoms by contributing to airway hyperresponsiveness and inflammation. Conversely, lower airway diseases, such as asthma, can predispose individuals to OSA by altering airway mechanics and increasing pharyngeal collapsibility. This bidirectional relationship underscores the need for integrated management approaches targeting both upper and lower airways to improve patient outcomes (Prasad et al., 2020; Taille et al., 2016).

While bronchodilator and anti‐inflammatory medications are the most common treatment options for asthma, nocturnal asthma symptoms are still common in 40% of individuals with asthma, especially those with co‐existing OSA, suggesting that other factors may contribute to asthma worsening at night. Deep breathing is an effective bronchodilator, which helps to release airway smooth muscles (ASM) and open closed airways in the lung (Kamio et al., 1990; Kelly et al., 2012; Kelly et al., 2013; White & Bradley, 2013; Yadollahi et al., 2015). However, it was shown that in patients with severe asthma or during an asthma attack, deep inspirations may not be effective as bronchodilators, or even act as bronchoconstrictors (Kelly et al., 2012; Kelly et al., 2013; Yadollahi et al., 2015). Interestingly, when the pharyngeal airway collapses during apneas or hypopneas in patients with OSA, the respiratory events are usually terminated with deep inspirations post‐arousals. Therefore, it is important to investigate how deep inspirations contribute to the pathophysiology of airway narrowing in patients with co‐existing asthma and OSA, particularly in those who had a history of asthma attack (Teodorescu et al., 2012; Yadollahi et al., 2015).

The goal of this study was to explore the patterns of deep inspirations that occur post termination of respiratory events in patients with OSA, with and without asthma. Since the impact of deep inspirations is less understood in those with asthma, we investigated deep inspirations in two cases of participants with or without asthma. This approach may offer new insights into the relationship between OSA, asthma, and the effectiveness of deep breathing in managing respiratory events in patients with OSA and asthma.

## 2 MATERIALS AND METHODS

### 2.1 Study participants

We analyzed data from the Sleep Heart Health Study (SHHS), which includes polysomnography from 5804 participants (Quan et al., 1997; Zhang et al., 2018). Apneas and hypopneas were scored based on guidelines from the American Academy of Sleep Medicine (Berry et al., 2012; Iber, 2007). Apneas were defined as periods of drop in breathing to <10% of normal breathing, lasting for more than 10 s, and hypopneas were defined as more than 30% reductions in airflow, lasting for more than 10 s and being associated with an arousal from sleep, or a minimum of 3% drops in oxygen saturation levels. Apnea‐hypopnea index (AHI) was defined as the number of apneas and hypopneas per hour of sleep. Individuals with AHI >5 were included as having OSA. OSA severity was categorized as mild (5< AHI ≤15), moderate (15 <AHI ≤30), or severe (AHI >30).

We included individuals with OSA without asthma (control group) or those who had experienced an asthma attack (asthma group). Asthma was assessed through self‐reported questionnaires at enrollment. Specifically, asthma status and the history of asthma attacks were determined using two questions: (1) “Has a doctor ever told you that you have asthma?” and (2) “Have you had an asthma attack at any time in the last 12 months?” (Quan et al., 1997; Zhang et al., 2018). For the asthma group, individuals were required to answer ‘Yes’ to both questions, indicating both a prior diagnosis of asthma and a history of asthma attacks within the last year. The control group was defined as individuals who answered ‘No’ to both questions, indicating no history of asthma or asthma attacks.

Participants were selected using a matched‐pair case–control design, where individuals in the asthma and control groups were paired one‐to‐one based on age, sex, race, body mass index (BMI), and OSA severity. Specifically, for age and BMI, controls were chosen to match asthma participants with a maximum age difference of 10 years. BMI categories were used to ensure a close match between the groups. We classified participants according to standard obesity classification categories: underweight (BMI < 18.5), normal weight (BMI ϵ [18.5, 25]), overweight (BMI ϵ [25, 30]), and obese (BMI > 30). For each asthma participant, a matched control was selected from the same BMI category.

### 2.2 Model development

The purpose of this section is to establish a systematic framework for modeling deep breaths after respiratory events and enabling comparison across groups. Our approach involved the following steps: (1) extracting post‐event deep breathing (PEDB) amplitudes from airflow signals as the average of the first three post‐event deep inspirations, (2) constructing a PEDB time series for each participant, (3) normalizing curve lengths across participants to allow averaging, (4) deriving representative PEDB curves for each group, (5) selecting the optimal polynomial order using error metrics, (6) assessing generalizability using train–test validation, where models are trained on one subset of data and evaluated on unseen data to ensure they captured physiological patterns rather than noise. This validation approach ensures that the observed group differences in PEDB curves are not artifacts of statistical modeling but instead reflect underlying physiological processes. This pipeline ensured that group‐level PEDB curves were standardized, reproducible, and physiologically interpretable.

**FIGURE 1:**
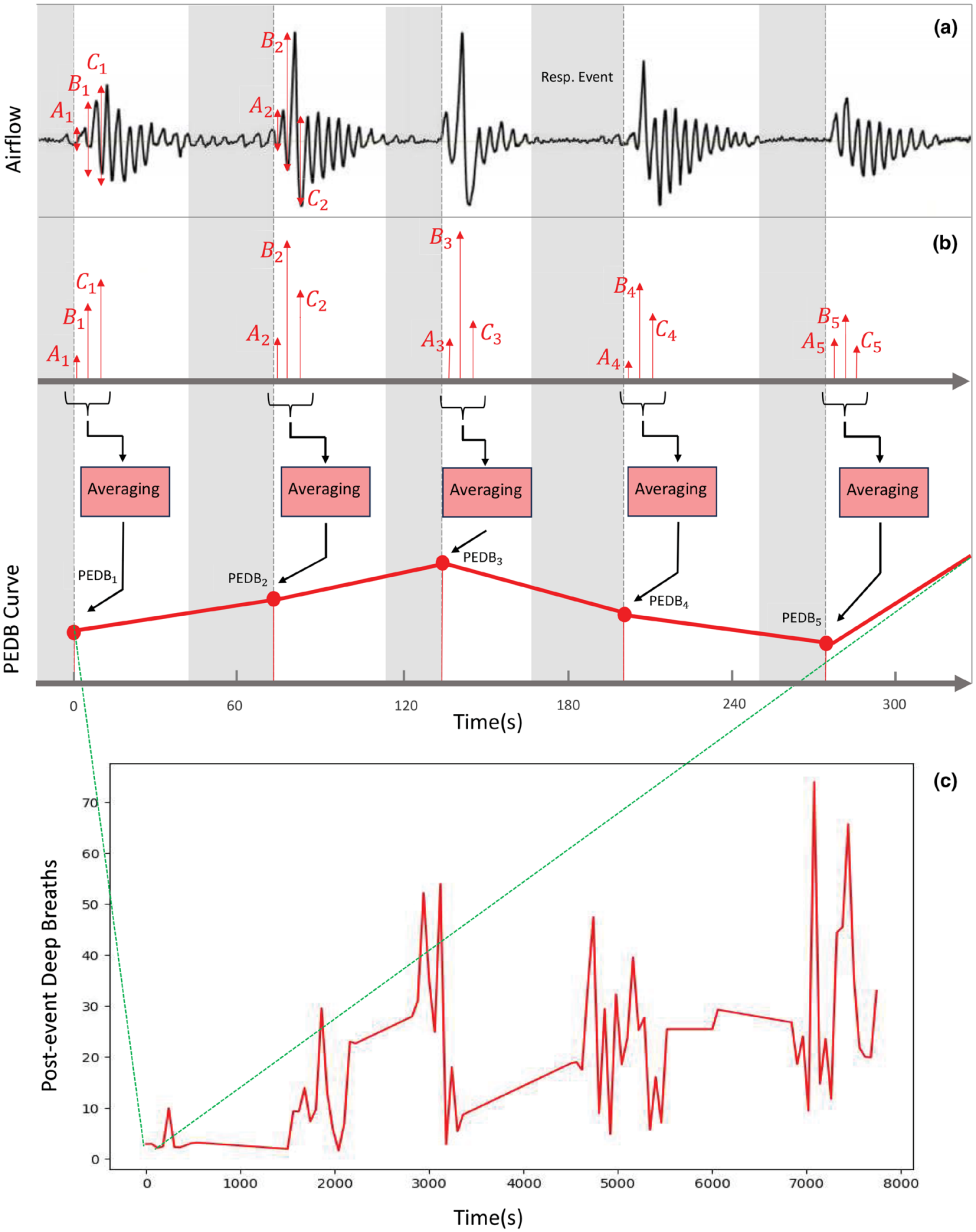
Example of Post‐event Deep Breathing (PEDB) curve calculation in an individual with AHI of 12 and no asthma. (a) The airflow signal and annotated respiratory events as shown by gray areas. $A_{1}$, $B_{1}$, and $C_{1}$ show three deep breath peak‐to‐peak absolute values related to the first post‐event overshoot of the airflow signal. (b) The average of $A_{i}$, $B_{i}$, and $C_{i}$ (i = 1, …, N and N = number of respiratory events for a sample patient) make the post‐event deep breathing value (${\text{PEDB}}_{i}$). Successive ${\text{PEDB}}_{i}$ related to consecutive respiratory events made the PEDB curve for each individual. (c) The PEDB‐time curve of an individual, whose start and end points are determined by the first and last respiratory event during sleep. Resp. Event: Respiratory Event.

Following most apneas or hypopneas, an overshoot in the airflow signal is typically observed, often associated with arousal or increased ventilatory drive due to an elevated loop gain (Sands et al., 2016). Modeling the deep inspirations that occur immediately after the termination of respiratory events is our objectives. Visual inspection of the airflow signals showed that the most pronounced overshoots consistently occurred within the first three breaths after an event, whereas subsequent breaths damped and resumed a stable rhythm. Therefore, we calculated the average peak‐to‐peak amplitude of these first three breaths as a representative measure of the post‐event deep inspirations (Figure 1a). We generated a new data series where each point i of the series indicates average of the peak‐to‐peak amplitude of three breaths after an apnea or hypopnea (Figure 1b and Equation (1)). For each participant, we used linear regression model to fit a curve to the PEDB time‐series (Equation (2)). The first and last data points in the PEDB curve indicate the first and last respiratory events of the participant during the night.

(1) $${\text{PEDB}}_{i}=\frac{1}{3}\;\sum_{j=1}^{3}\left(\max\left(F_{i,j}\right)-\min\left(F_{i,j}\right)\right)=\frac{1}{3}\;\left(A_{i}+B_{i}+C_{i}\right)$$

where $F_{i,j}$ is the airflow signal for $j^{\mathrm{th}}$ breath after the $i^{\mathrm{th}}$ respiratory event and $A_{i},B_{i},C_{i}$ are the peak‐to‐peak amplitudes of the first, second, and third post‐event deep breaths, respectively. The PEDB time series for a given participant is then defined as:

(2) $$\text{PEDB}=\left\{{\text{PEDB}}_{1},{\text{PEDB}}_{2},\ldots ,{\text{PEDB}}_{N}\right\}$$

where *N* is the total number of respiratory events recorded for that participant.

Since the number of respiratory events $N_{k}$ varied across participants *k*, polynomial interpolation of first and second degrees was used to standardize the length of all PEDB time series. This ensured that all curves had the same number of data points across participants, allowing for meaningful averaging within groups. We rescaled each participant's PEDB time series to match the maximum curve length M observed in their respective group (control, asthma, or OSA subgroups).

(3) $$M=\max_{k}\;\left(N_{k}\right)$$

Let ${\text{PEDB}}_{i}$ be the original PEDB value for the $i^{\mathrm{th}}$ respiratory event, where i=1,2,…,N. We define the normalized position of each original data point as:

(4) $$x_{i}=\frac{i-1}{N-1},\;i=1,2,\ldots ,N,\;x_{i}\;\epsilon \;\left[0.1\right]$$

A polynomial interpolation function $P\left(x\right)$ of degree one or two is fitted such that it exactly matches the original PEDB time series:

(5) $$P\left(x_{i}\right)={\text{PEDB}}_{i},\;\forall i\;\epsilon \;\left\{1,\ldots ,N\right\}$$

We then evaluate P(*x*) at M uniformly spaced points in the interval [0.1], given by:

(6) $$x_{j}^{\prime }=\frac{j-1}{M-1},\;j=1,2,\ldots ,M$$

This yields the standardized PEDB time series for each participant:

(7) $$\tilde{\text{PEDB}}=\left\{P\left(x_{1}^{\prime }\right),P\left(x_{2}^{\prime }\right),\ldots ,P\left(x_{M}^{\prime }\right)\right\}$$

Finally, within each participant group, the characteristic PEDB curve is computed as the mean of the standardized PEDB curves from all K participants in the group:

(8) $$\bar{\text{PEDB}}=\frac{1}{K}\;\sum_{k=1}^{K}{\tilde{\text{PEDB}}}^{\left(k\right)}$$

To smooth the characteristic PEDB curve, polynomial regression models of degrees one to three were applied. To estimate the confidence intervals for the characteristic curves within each group, we applied bootstrapping with 1000 samples to the PEDB time series to generate resampled datasets.

To determine the optimal polynomial order for interpolation and curve fitting, we conducted a systematic evaluation using both quantitative and qualitative metrics. The Root Mean Square Error (RMSE) was calculated to quantify the deviation between the fitted curves and the actual data points. Given the true datapoints $y_{j}$ = ${\bar{\text{PEDB}}}_{j}$ and fitted values ${\hat{y}}_{j}=\hat{\text{PEDB}}\left(x_{j}\right)$, RMSE is computed as:

(9) $$\text{RMSE}=\sqrt{\frac{1}{M}\sum_{j=1}^{M}{\left({\hat{y}}_{j}-y_{j}\right)}^{2}}$$

To evaluate generalizability, we performed cross‐validation by dividing the dataset into training (80%) and testing (20%) subsets. Regression models were fitted on the training data and evaluated on the test data. High‐order polynomial regression may result in overfitting, characterized by complex and oscillatory curves. Overfitting is indicated by a low RMSE on the training set but a high RMSE on the test set, along with noticeable oscillations. In contrast, underfitting yields high RMSE values and excessively smooth curves. The optimal model achieves a balance between low RMSE and appropriate curve smoothness.

All signal processing and analysis were implemented in Python. To ensure transparency and reproducibility, the source code is available at https://github.com/sshmousavi/OSA‐Asthma‐DI‐Analysis.

### 2.3 Curves distance measurement

To compare the distance between characteristic curves of asthma and control groups across all OSA severity levels, we used the Dynamic Time Warping (DTW) method. DTW is an algorithm designed to assess the similarity between two temporal sequences, such as time series (Sakoe & Chiba, 1978; Varatharajan et al., 2018). It seeks an optimal alignment between the given sequences, subject to specific constraints: matching indices from the first sequence to one or more indices from the second sequence, ensuring a monotonically increasing mapping of indices, and connecting the first and last indices of both. The optimal alignment minimizes the cost, which is computed as the quadratic distance between matched indices. This distance measure is particularly effective at penalizing significant deviations. $D\;\left(i,j\right)$ as the accumulated cost at point $\left(i,j\right)$ can be calculated as follow:

(10) $$D\;\left(i,j\right)=|x_{i}-y_{j}|+\min\left(D\left(i-1,j\right),D\left(i,j-1\right),D\left(i-1,j-1\right)\right)$$

where $x=\left[x_{1},x_{2},\ldots ,x_{L}\right]\;\text{and}\;y=\left[y_{1},y_{2},\ldots ,y_{R}\right]$ are two time series. The final DTW distance is given by:

(11) $$\mathrm{DTW}\;\left(x,y\right)=\sqrt{D\left(L,R\right)}$$

DTW is particularly effective for comparing sequences of varying lengths or those with nonlinear temporal alignments. Accordingly, we applied it prior to polynomial interpolation to measure the distance of time series with unmatched lengths. Unlike Euclidean and Manhattan distance, which only allow one‐to‐one point comparisons, DTW enables many‐to‐one alignments, effectively accounting for temporal distortions between sequences to achieve optimal matching.

### 2.4 Statistical analysis

Descriptive statistics of the demographic and polysomnographic characteristics were reported for control and asthma groups as mean ± SD for continuous data and as the number for categorical data. To compare the demographic parameters and sleep structure, we performed independent *t*‐tests for continuous data and Fisher's exact test for categorical data. To assess the potential temporal changes in the PEDB curves overnight, the PEDB curves were divided into four quartiles, and the averages in each quartile were calculated. The Wilcoxon Signed‐Rank test was used to compare within‐group changes in PEDB values across different quartiles. Between‐group changes in PEDB values were assessed using the Mann–Whitney *U* test. Statistical differences were considered significant for *p*‐value <0.05. To examine the relationship between respiratory event duration and the corresponding PEDB values, Spearman rank correlation coefficients (SRCs) were computed for each subject. These calculations were performed within subgroups defined by OSA severity in both the asthma and control groups.

## 3 RESULTS

### 3.1 Participant characteristics

A total of 202 individuals with OSA were included (101 participants as controls and 101 with asthma attack). The demographic characteristics of participants in each group are shown in Table 1. By design, for all participants and sub‐groups of OSA severities, age, sex, race, and BMI were similar between individuals in asthma and control groups (*p*‐value >0.1). Also, smoking, cardiovascular disease, chronic conditions, and stroke were also similar between all the participants in the groups (*p*‐value >0.1). In both groups, there were 68 participants with mild OSA, 19 with moderate, and 14 with severe OSA (Table 2). Statistical analysis also showed no significant differences between the two groups for the sleep measures (*p*‐value >0.1), suggesting the two groups are similar with respect to sleep structure and sleep apnea severity.

**TABLE 1:** Demographic characteristics of individuals in the dataset.

		Asthma				Control			
OSA severity (*n*)		All (101)	Mild (68)	Moderate (19)	Severe (14)	All (101)	Mild (68)	Moderate (19)	Severe (14)
Age, year		62 (10.7)	61.3 (12.1)	61.6 (8.0)	61.0 (11.6)	61.3 (11)	62.1 (11.8)	61.5 (7.4)	63.0 (12.3)
BMI (kg/m^2^)		30.9 (6.2)	31.0 (6.7)	29.0 (5.3)	31.1 (6.4)	30.6 (6.3)	31.1 (6.8)	27.9 (5.7)	31.3 (5.2)
Sex, *n* (%)	Male	31 (31)	14 (20)	9 (48)	8 (57)	31 (31)	14 (20)	9 (48)	8 (57)
	Female	70 (69)	54 (80)	10 (52)	6 (43)	70 (31)	54 (80)	10 (52)	6 (43)
Race, *n* (%)	White	89 (88)	64 (94)	13 (68)	12 (85)	89 (88)	64 (94)	13 (68)	12 (85)
	Black	12 (12)	4 (6)	6 (32)	2 (15)	12 (12)	4 (6)	6 (32)	2 (15)
Smoking, *n* (%)	Never	51 (50)	35 (51)	12 (63)	4 (29)	51 (50)	38 (55)	9 (48)	4 (29)
	Former	36 (36)	23 (33)	5 (26)	8 (57)	40 (40)	29 (42)	10 (52)	1 (7)
	Current	14 (14)	10 (16)	2 (11)	2 (14)	10 (10)	1 (1)	0 (0)	9 (64)
Cardiovascular disease, *n* (%)		18 (18)	9 (13)	6 (32)	3 (21)	10 (10)	6 (9)	2 (10)	2 (14)
COPD, *n* (%)		7 (7)	2 (3)	2 (10)	3	0	0	0	0
Stroke, *n* (%)		0	0	0	0	5 (5)	2 (3)	2 (10)	1 (7)

*Note*: The data are presented as average (SD).

Abbreviations: BMI, Body Mass Index; COPD. chronic obstructive pulmonary disease; OSA, obstructive sleep apnea.

**TABLE 2:** Sleep‐related characteristics of individuals in dataset.

		Asthma				Control			
OSA severity (*n*)		All (101)	Mild (68)	Moderate (19)	Severe (14)	All (101)	Mild (68)	Moderate (19)	Severe (14)
TST, *n*	≤5 h	21	11	8	2	22	10	6	6
	5–7 h	64	47	6	11	68	53	10	5
	>7 h	16	10	5	1	11	5	3	3
AHI, event/h		17.6 (15.4)	8.8 (5.5)	18.9 (6.9)	44.5 (13.5)	19.8 (15.8)	10.6 (8.1)	23 (11.6)	43.8 (9.6)
Obstructive AHI, event/h		17 (14.7)	8.6 (5.4)	18.6 (6.8)	42.2 (13.7)	19.4 (15.5)	10.4 (8.1)	22.8 (11.4)	43.8 (9.6)
Central AHI, event/h		10.6 (10.7)	6.7 (5.1)	11.8 (5.6)	21.5 (18.6)	13.1 (12.1)	7.8 (7.8)	15.1 (10.5)	27.4 (13.2)
REM AHI, event/h		24.5 (18.4)	27.8 (19.5)	40.9 (20.8)	41.1 (18.5)	26.2 (22)	28.4 (20.9)	38.2 (23.1)	50.3 (19.5)
NREM AHI, event/h		12.5 (14.3)	9.4 (7)	23.2 (8.9)	46.5 (13.7)	15.9 (15.7)	11.8 (9)	29.4 (18.7)	51.2 (9.1)
Arousal Index^a^		23.8 (12.5)	19.8 (9.4)	23.5 (11.8)	36.5 (13.7)	23.7 (12.2)	19.3 (9.3)	30.9 (13)	29.3 (12.9)
Respiratory event duration, s		22.1 (9.4)	20.9 (9.5)	20.8 (8.5)	25.4 (10.2)	22.5 (10)	21.4 (8.9)	21.8 (10.6)	24.5 (10.5)
Sleep efficiency^b^, %		80.6 (11.1)	81.6 (8.8)	79.2 (14.1)	79.3 (13.1)	81.9 (11.3)	82.2 (11.6)	82.0 (12.6)	80.6 (8.9)
Supine sleep ratio^c^, %		32.3 (32.8)	37.8 (34.1)	24.0 (37)	38.7 (31.8)	32 (33.2)	33.8 (31.2)	18.7 (26.7)	31.2 (35.5)

*Note*: The data are presented as average (SD).

Abbreviations: AHI, apnea‐hypopnea index; NREM, Non‐rapid eye movement; REM, rapid eye movement; TST, total sleep time.

^a^ Total number of arousals per hour of sleep.

^b^ The ratio of sleep duration to in‐bed period.

^c^ Percentage of total sleep duration in the supine position.

### 3.2 Post‐event deep breathing model

**FIGURE 2:**
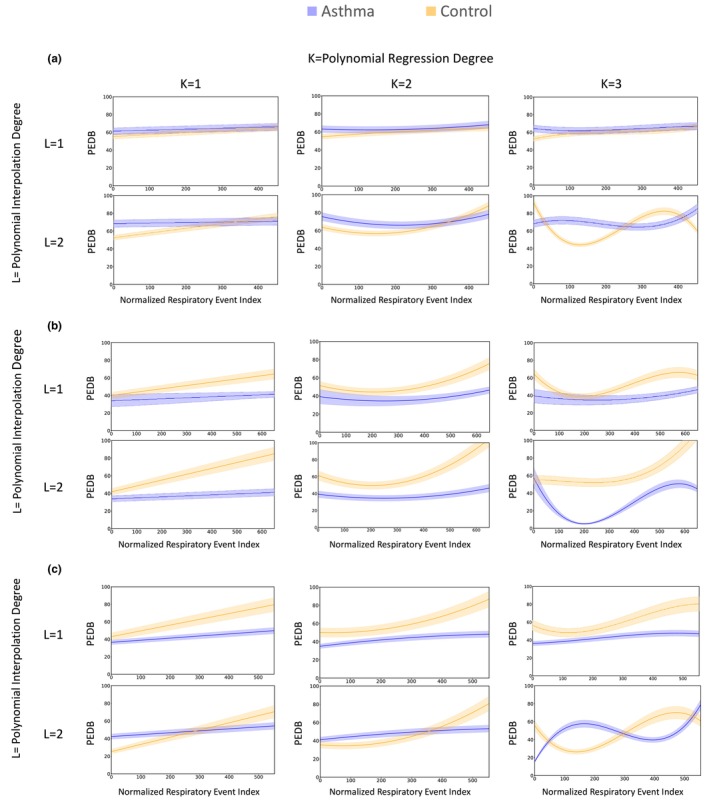
Illustration of post‐event deep breathing (PEDB) curves for two groups of OSA patient, with asthma (blue) and without asthma (orange), using polynomial interpolation of first and second degree and regression of degrees one to three, across three OSA severity levels (mild, moderate, severe) throughout total sleep time. Degrees of polynomials for interpolation and regression are indicated by L and K, respectively. The horizontal axis shows the normalized respiratory event index (a rescaled sequence of respiratory events), and the vertical axis indicates PEDB values. Shaded areas represent 95% confidence intervals. Selecting the optimal polynomial degree involves balancing root mean square error and curve smoothness. (a) Mild OSA. (b) Moderate OSA. (c) Severe OSA.

**FIGURE 3:**
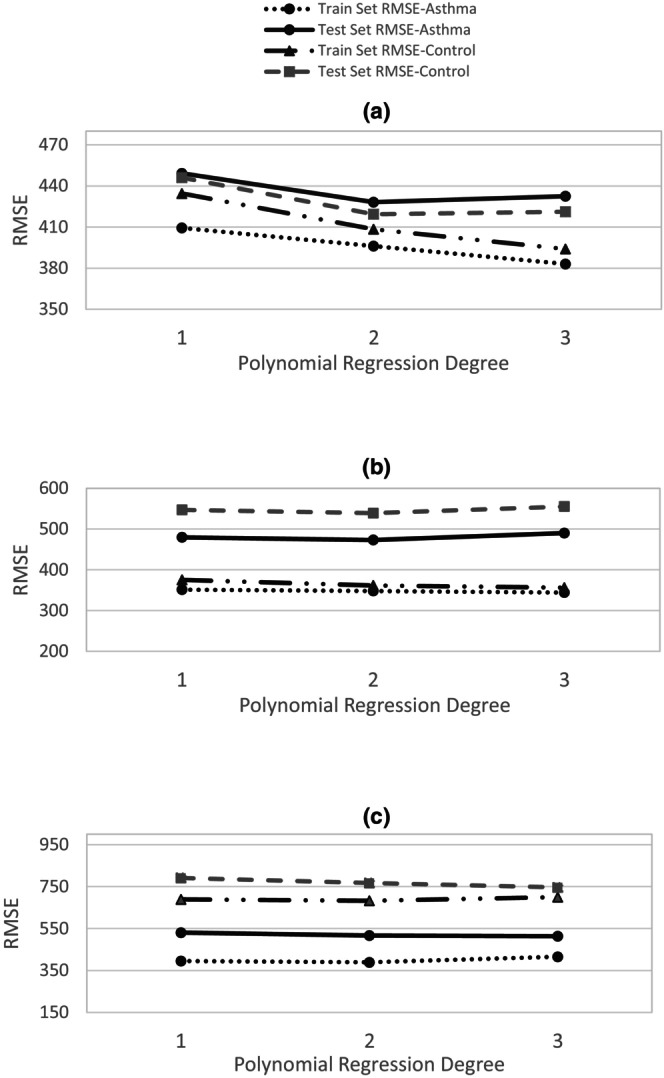
Root Mean Square Errors (RMSE) of training and testing sets for two groups of OSA patients, with and without asthma, across polynomial curve fitting of degrees one to three, considering three OSA severity levels. The training and testing sets were split in an 80–20 ratio prior to applying the polynomial interpolation method. (a) Mild OSA. (b) Moderate OSA. (c) Severe OSA.

Figure 2 shows the PEDB curves for both asthma and control groups during sleep, obtained through first and second order polynomial interpolation and polynomial regression of degrees 1 to 3, across three levels of OSA severity, and Figure 3 presents the corresponding RMSE values for the training and testing sets for each OSA severity level (severe, moderate, and mild).

Based on the RMSE results, the error on the test set decreases up to degree 2 of polynomial regression and then increases at degree 3, suggesting that degree 2 offers the best generalization (Figure 3). Additionally, degree 2 yields smoother curves compared to degree 1, while degree 3 introduces overfitting‐related oscillations. Therefore, degree 2 was selected as the optimal degree for polynomial regression. For interpolation, however, the second‐order polynomial introduced oscillatory artifacts, whereas the first‐order polynomial (linear interpolation) produced the most stable curves and was therefore considered the most appropriate choice.

**FIGURE 4:**
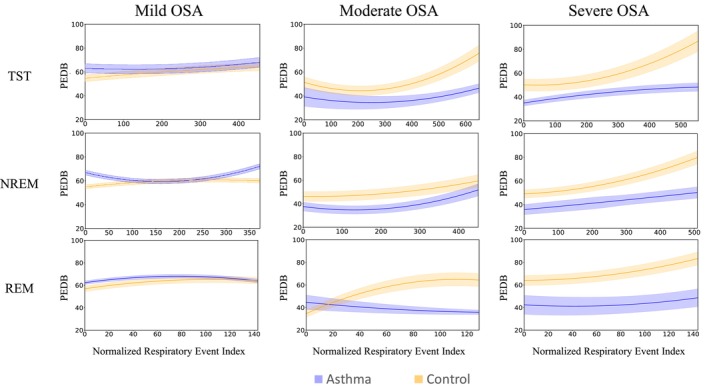
The characteristic Post‐event Deep Breathing (PEDB) curves of two groups of OSA patients with asthma (blue) and without asthma (orange), considering three OSA severity levels (mild, moderate, and severe) across total sleep time (both NREM and REM stages) as well as REM and NREM stages separately. The horizontal axis represents the normalized respiratory event index, which corresponds to a rescaled sequence of number of respiratory events. The vertical axis indicates post‐event deep breathing values. Shaded regions show the 95% confidence interval. NREM, non‐rapid eye movement; REM, rapid eye movement; TST, total sleep time.

Figure 4 shows the PEDB characteristic curves for the control and asthma groups, during total sleep, NREM, and REM sleep. Based on the maximum number of respiratory events for mild, moderate, and severe OSA groups, the normalized length of PEDB characteristic curves was 455, 650, and 555 during total sleep time (TST), 372, 450, and 509 during NREM sleep, and 144, 130, and 145 during REM sleep, respectively. For individuals with moderate or severe OSA, the patterns of the PEDB curves were different between the asthma and control groups, particularly during the last quartile of sleep (Figure 4). Interestingly, while the PEDB characteristic curves remained flat for individuals with asthma for various OSA severities and sleep stages, in the control group, the PEDB curves increased overnight (larger deep inspirations compared to the beginning of the night), particularly for the control group with severe OSA.

**FIGURE 5:**
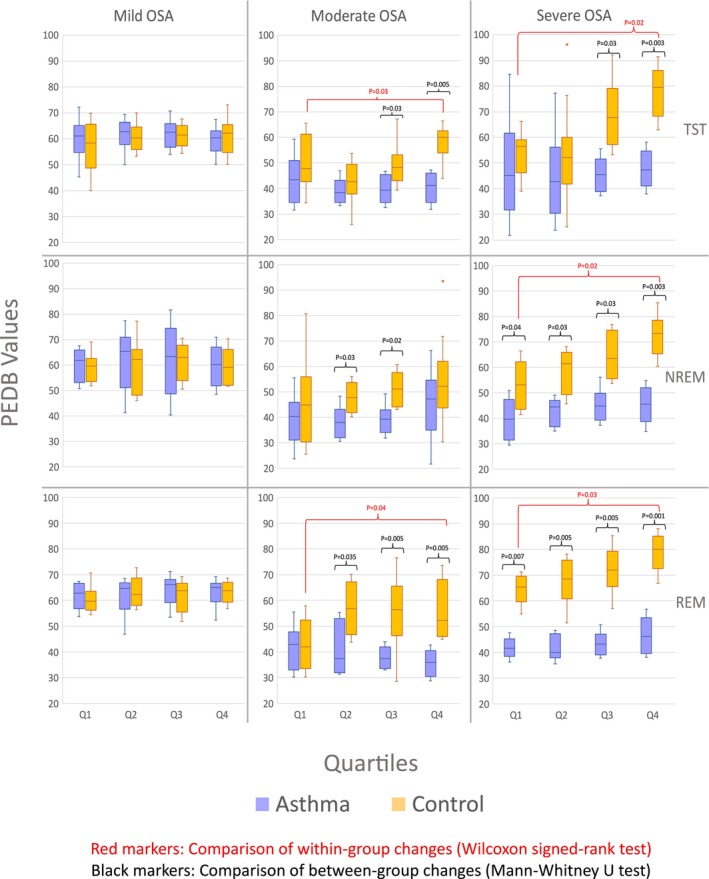
The average post‐event deep breathing (PEDB) values in the asthma group (blue box plots) and the non‐asthma group (orange box plots) across all quartiles, sleep stages, and OSA severities. Within‐group changes were assessed using the Wilcoxon signed‐rank test (red markers), and between‐group differences were assessed using the Mann–Whitney U test (black markers). Significant *p*‐values are indicated on the plots. NREM, non‐rapid eye movement; OSA, obstructive sleep apnea; PEDB, post‐event deep breaths; REM, rapid eye movement; TST, total sleep time.

These results are confirmed by statistical analyses of PEDB values in different quartiles of the PEDB characteristic curves for individuals in the asthma and control groups (Figure 5). In the control group, for individuals with moderate and severe OSA, the magnitude of PEDB increased significantly from the first quartile to the fourth quartile. On the other hand, there were no significant changes in PEDB values overnight for the control group with mild OSA or the asthma group (independent of OSA severity). When comparing between asthma and control groups, for individuals with moderate or severe OSA, the PEDB values are larger in the control group compared to the asthma group, indicating larger deep inspirations post respiratory events.

**FIGURE 6:**
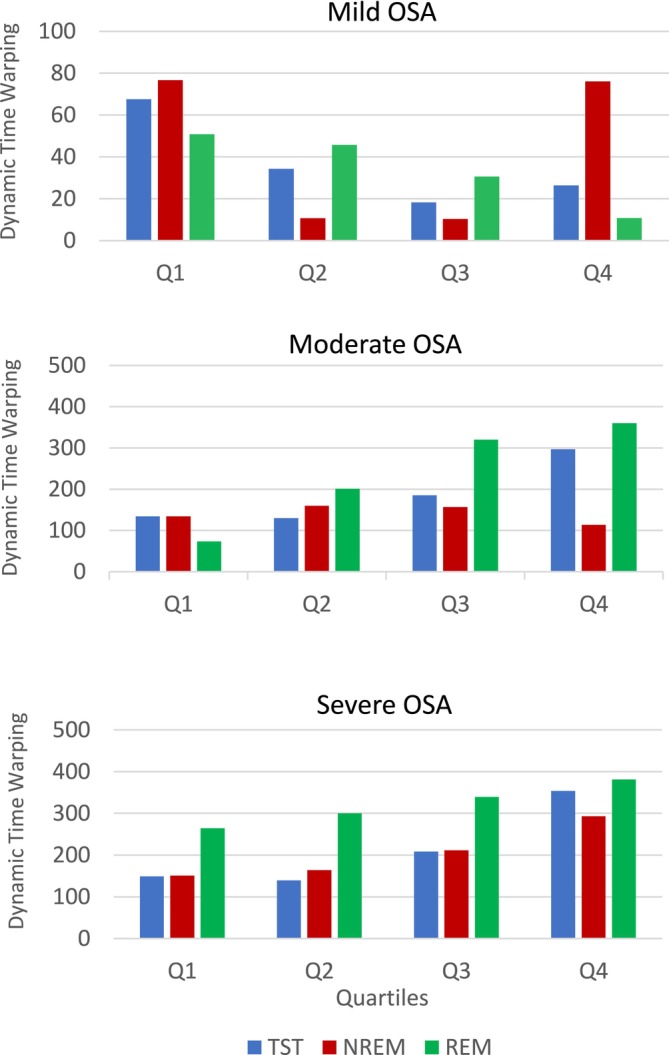
Dynamic Time Warping values, calculated between asthma attacks and without asthma individuals' characteristic post‐event deep breathing curves in all quartiles across different sleep stages and OSA severities.

DTW values further support these findings by quantifying the distance between PEDB curves of the asthma and control groups across all OSA severity levels and sleep stages (Figure 6). For individuals with severe OSA, DTW values increased from the first to the fourth quartile, indicating a growing separation between the two curves throughout the night. In moderate OSA, this increasing trend was particularly evident during TST and REM stages.

Our analysis showed that respiratory event durations were similar between asthma and control groups (Table 2), indicating that the larger value of deep inspirations observed in the control group was not dependent on event severities. Across all subgroups, the SRCs were centered near zero, indicating no consistent monotonic relationship between event duration and PEDB within individuals. In the asthma group, the median (interquartile range) SRCs were 0.0008 (−0.08 to 0.09) in mild OSA, −0.01 (−0.06 to 0.04) in moderate OSA, and 0.0001 (−0.05 to 0.06) in severe OSA. Similar distributions were observed in the control group.

## 4 DISCUSSION

This is the first study to comprehensively investigate the patterns of deep inspirations post respiratory events in patients with OSA and a history of asthma attack versus those without asthma. Our study has three important novelties: (1) since we have performed one‐on‐one matching between individuals in the asthma and control groups, age, sex, BMI, race, OSA severity, and sleep structure were similar in both groups. This strong design reduces the impact of confounding factors and improves the reliability of our results. (2) Across all OSA severity levels, in patients with a history of asthma attack, the peak‐to‐peak amplitude of deep inspirations post‐respiratory events remained similar throughout the sleep. In contrast, in those without asthma, an overnight increase in peak‐to‐peak amplitude of deep inspirations post‐respiratory events was observed in individuals with moderate and severe OSA. (3) The amplitude of deep inspirations post respiratory events was significantly smaller in the asthma group compared to the control group, specifically, in those with moderate and severe OSA.

The matching of asthma and control groups based on key demographic and clinical factors such as age, sex, BMI, race, and OSA severity is critical for minimizing the potential confounding factors, such as sleep structure, chronic conditions such as heart or other lung disease that may impact interaction between asthma and OSA, or respiratory patterns. By controlling for these variables, the study ensures that observed differences in deep inspiration amplitudes were more likely reflect the influence of asthma rather than confounding factors. This methodological approach strengthens the validity of the findings and provides a more accurate representation of the physiological differences between individuals with and without asthma attacks in the context of OSA.

An important finding of our study was that in controls without asthma, the amplitude of deep inspirations post respiratory events increased overnight, but it did not change in those with a history of asthma attack. To our knowledge, this is the first study to investigate the changes in deep inspirations post arousals during sleep in asthma patients. While some previous studies have examined the magnitude of post‐event ventilations in relation to hypercapnia, they primarily excluded asthma patients and focused on subjects with obesity hypoventilation syndrome (Jaimchariyatam et al., 2013; Kittivoravitkul et al., 2016). In controls without asthma, the increase in deep inspirations may represent more severe pharyngeal airway obstructions in the later part of sleep compared to the beginning of sleep. Increases in pharyngeal airway severity throughout the night may be due to circadian changes in respiratory control, such as increases in vagal tone and the consequent bronchoconstriction in the early morning (Sato et al., 2021; Sorkness et al., 2011), changes in sleep structure and less deep sleep episodes toward the end of sleep, and increases in inflammatory markers in the morning (Mat et al., 2021; Sato et al., 2021). Another potential mechanism could be the gravity‐dependent shift of fluid from lower extremities to thorax and neck during sleep, which occurs progressively throughout the night and could narrow pharyngeal airways and worsen respiratory events (Almendros et al., 2007; Boyd et al., 2004; Crisford et al., 2021). This complies with our previous study which has shown that snoring sounds intensity increase from the first to the second half of sleep (Saha et al., 2024).

Since OSA severity and sleep structure were similar in patients with a history of asthma attack and control groups, the lower amplitude of deep inspirations in the asthma group may indicate more lower airway narrowing in patients with a history of asthma attack than the control group, and particularly in the latter part of sleep. While similar to the control group, circadian changes in lung volume, vagal tone, and respiratory control could narrow lower airways in patients with asthma, the impact of circadian variations in cytokine and hormone secretion on lower airway narrowing may be more prominent in patients with asthma than controls (Sato et al., 2021; Sorkness et al., 2011). Inflammatory markers peak in the morning (Mat et al., 2021; Sato et al., 2021). Inflammatory mediators dilate blood vessels, increasing fluid leakage and further narrowing airways. This nighttime inflammation particularly thickens airway walls, limiting dilation and reducing airflow to the alveoli (Prasad et al., 2014; Prasad et al., 2020). Although airway resistance was not directly measured in our study, our findings are consistent with previous studies where continuous measurements demonstrated higher resistance and lower variability in airway resistance in patients with asthma compared to non‐asthmatic controls during sleep (Guilleminault et al., 1993). Consequently, asthma patients commonly experience more frequent symptoms in the morning compared to nighttime (Mat et al., 2021; Sato et al., 2021). Future studies can specifically focus on measuring airway resistance to verify these mechanisms. Also, we have shown that in patients with asthma and moderate to severe OSA, pharyngeal airway obstructions increase negative intrathoracic pressure, promoting fluid shifts into the thorax, and further narrowing lower airways (Cao et al., 2022; Cao et al., 2024; Chavoshian et al., 2023; Montazeri Ghahjaverestan et al., 2024). Future studies should investigate the physiological mechanisms that may contribute to different patterns of deep inspirations in those with OSA, with and without a history of asthma attack.

Deep inspirations are recognized as potent bronchodilators in a healthy lung (Kelly et al., 2012; Kelly et al., 2013; White & Bradley, 2013). However, their efficacy is compromised in asthmatic lungs and may even be reversed during spontaneous asthma attacks (Guilleminault et al., 1993; Teodorescu et al., 2012; Yadollahi et al., 2015). The beneficial bronchodilation effect of breathing and deep inspiration is credited to the stretching that fluidizes the ASM cell, reducing its contractile force (Krishnan et al., 2009; Krishnan et al., 2012; Lavoie et al., 2012; Sheel et al., 2009; Teodorescu et al., 2009; Trepat et al., 2007; Wang et al., 2017). In maintaining airway patency, the dynamic act of breathing is crucial, particularly in the face of significant ASM activation (Brightling et al., 2016; Gupta et al., 2010; Thomson et al., 2015; Wang et al., 2017). However, during an asthmatic attack, this positive feedback mechanism can break down (Brown et al., 2017; Carroll et al., 2015; Eddy et al., 2020; Thomson et al., 2015). This breakdown is associated with increased ASM contractile stimulus, greater ASM mass due to fluid shifts and inflammation (Kelly et al., 2012; Kelly et al., 2013; Prasad et al., 2014; Yadollahi et al., 2015), and decreased lung parenchymal tethering forces due to elevated airway wall thickness and reduced lung recoil (Galbán et al., 2012; Guo et al., 2017; Svenningsen et al., 2013; Thomson et al., 2015; Vasilescu et al., 2019). Our results for reduced amplitude of deep inspirations in patients with asthma and moderate–severe OSA, especially later in the sleep, may imply potential reductions in the bronchodilator effect of deep inspirations (Yadollahi et al., 2015). This may indicate the diminished role of deep inspiration as a potential contributor to the higher frequency of asthma symptoms closer to morning compared to earlier in the night.

Medication use, especially corticosteroids, can affect respiratory physiology. However, in our dataset, most participants with asthma were not using inhaled or oral steroids during the study, and none of the control participants used these medications. Therefore, our results probably reflect group differences that are not mainly caused by corticosteroid use.

This study has some limitations. Asthma attacks were not assessed through standard clinical tests; instead, their occurrence was determined based on self‐reported questionnaires. Additionally, the study was not conducted during active asthma attacks but rather relies on the patient's history of asthma episodes. The use of thermistor‐based signals for airflow amplitude measurement, which provides a qualitative assessment, is another limitation of this study. Unlike nasal pressure measurements, thermistors detect airflow through temperature changes, which may introduce some variability due to factors such as mouth breathing and environmental conditions. While this method remains a widely used approach for airflow monitoring, slight variations in amplitude should be considered when interpreting the results. Our analysis focused on extreme conditions, comparing individuals with a history of asthma attack to those without asthma, without accounting for varying severities of asthma. Addressing these factors and expanding the study to include different asthma severity levels will be the focus of future research.

## 5 CONCLUSION

In conclusion, this study is the first to explore how deep inspirations following respiratory events differ between individuals with and without a history of asthma attack across OSA severity levels. Our findings provide strong evidence that patients with severe OSA and a history of asthma attack exhibit reduced deep inspiration amplitudes compared to those with OSA but no asthma. This dampened response may limit their ability to benefit from the bronchodilatory effects of deep inspirations. Additionally, it is possible that, beyond mechanical limitations, altered airway responsiveness or a tendency toward bronchoconstriction in asthmatic individuals may also contribute to the observed differences. These results highlight the need for further studies to investigate the mechanisms behind impaired deep inspiration in this population, which could improve our physiological understanding of lung function in patients with co‐existing asthma and OSA.

## AUTHOR CONTRIBUTIONS

**Shokoufeh Mousavi**: Conceived and designed research, analyzed data, performed experiments, interpreted results of experiments, prepared figures, drafted manuscript, edited, and revised the manuscript, approved final version of the manuscript. **Maryam Mohebbi**: Conceived and designed research, analyzed data, performed experiments, interpreted results of experiments, edited, and revised the manuscript, approved final version of the manuscript. **Parisa Adimi Naghan**: Conceived and designed research, interpreted results of experiments, edited, and revised the manuscript, approved final version of the manuscript. **Azadeh Yadollahi**: Conceived and designed research, analyzed data, performed experiments, interpreted results of experiments, edited, and revised the manuscript, approved final version of the manuscript.

## FUNDING INFORMATION

This work has been supported by the Center of International Scientific Studies and Collaborations (CISSC), Ministry of Science, Research and Technology of Iran.

## CONFLICT OF INTEREST

No conflicts of interest, financial or otherwise, are declared by the authors.

## ETHICS STATEMENT

The analyses in this study were performed using de‐identified, publicly available data from the Sleep Heart Health Study (SHHS; ClinicalTrial.gov identifier: NCT00005275). As all data were previously collected with informed consent and released for public research use, additional ethical approval was not required for this study.

## Data Availability

This study used data from the Sleep Heart Health Study (SHHS), a publicly available dataset. The SHHS dataset can be accessed at https://sleepdata.org/datasets/shhs. Researchers can obtain the data by following the access procedures outlined on the website. The Sleep Heart Health Study (SHHS) was supported by National Heart, Lung, and Blood Institute cooperative agreements U01HL53916 (University of California, Davis), U01HL53931 (New York University), U01HL53934 (University of Minnesota), U01HL53937 and U01HL64360 (Johns Hopkins University), U01HL53938 (University of Arizona), U01HL53940 (University of Washington), U01HL53941 (Boston University), and U01HL63463 (Case Western Reserve University). The National Sleep Research Resource was supported by the National Heart, Lung, and Blood Institute (R24 HL114473, 75N92019R002).

## References

[phy270622-bib-0001] Ahookhosh, K. , Pourmehran, O. , Aminfar, H. , Mohammadpourfard, M. , Sarafraz, M. M. , & Hamishehkar, H. (2020). Development of human respiratory airway models: A review. European Journal of Pharmaceutical Sciences, 145, 105233. 31978589 10.1016/j.ejps.2020.105233

[phy270622-bib-0002] Almendros, I. , Acerbi, I. , Puig, F. , Montserrat, J. M. , Navajas, D. , & Farré, R. (2007). Upper‐airway inflammation triggered by vibration in a rat model of snoring. Sleep, 30(2), 225–227. 17326549 10.1093/sleep/30.2.225

[phy270622-bib-0003] Berry, R. B. , Budhiraja, R. , Gottlieb, D. J. , Gozal, D. , Iber, C. , & Kapur, V. K. (2012). Rules for scoring respiratory events in sleep: update of the 2007 AASM manual for the scoring of sleep and associated events: deliberations of the sleep apnea definitions task force of the American Academy of sleep medicine. Journal of Clinical Sleep Medicine, 8(5), 597–619. 23066376 10.5664/jcsm.2172 PMC3459210

[phy270622-bib-0004] Boyd, J. H. , Petrof, B. J. , Hamid, Q. , Fraser, R. , & Kimoff, R. J. (2004). Upper airway muscle inflammation and denervation changes in obstructive sleep apnea. American Journal of Respiratory and Critical Care Medicine, 170(5), 541–546. 15151922 10.1164/rccm.200308-1100OC

[phy270622-bib-0005] Brightling, C. E. , Nordenmark, L. H. , Jain, M. , Piper, E. , She, D. , Braddock, M. , Colice, G. , & Tornling, G. (2016). Effect of anti–IL‐13 treatment on airway dimensions in severe asthma. American Journal of Respiratory and Critical Care Medicine, 194(1), 118–120. 27367889 10.1164/rccm.201511-2224LE

[phy270622-bib-0006] Brockmann, P. E. , Bertrand, P. , & Castro‐Rodriguez, J. A. (2014). Influence of asthma on sleep disordered breathing in children: a systematic review. Sleep Medicine Reviews, 18(5), 393–397. 24629825 10.1016/j.smrv.2014.01.005

[phy270622-bib-0007] Brown, R. H. , Henderson, R. J. , Sugar, E. A. , Holbrook, J. T. , & Wise, R. A. (2017). Reproducibility of airway luminal size in asthma measured by HRCT. Journal of Applied Physiology, 123(4), 876–883. 28705995 10.1152/japplphysiol.00307.2017 PMC5668456

[phy270622-bib-0008] Cao, X. , de Oliveira Francisco, C. , Bradley, T. D. , Montazeri Ghahjaverestan, N. , Tarlo, S. M. , Stanbrook, M. B. , Chapman, K. R. , Inman, M. , & Yadollahi, A. (2022). Association of obstructive apnea with thoracic fluid shift and small airways narrowing in asthma during sleep. Nature and Science of Sleep, 14, 891–899. 10.2147/NSS.S359021 PMC9091700 35573055

[phy270622-bib-0009] Cao, X. , Francisco, C. O. , Bhatawadekar, S. A. , Makanjuola, J. , Tarlo, S. M. , Stanbrook, M. B. , Inman, M. D. , & Yadollahi, A. (2024). A pilot study to assess the effects of preventing fluid retention in the legs by wearing compression stockings on overnight airway narrowing in mild asthma. Sleep and Breathing, 28, 1285–1292. 38365985 10.1007/s11325-023-02976-0

[phy270622-bib-0010] Carroll, J. D. , Magnussen, J. , Berend, N. , Salome, C. , & King, G. G. (2015). Greater parallel heterogeneity of airway narrowing and airway closure in asthma measured by high‐resolution CT. Thorax, 70, 1163–1170. 26354711 10.1136/thoraxjnl-2014-206387

[phy270622-bib-0011] Castro‐Rodriguez, J. A. , Brockmann, P. E. , & Marcus, C. L. (2017). Relation between asthma and sleep disordered breathing in children: is the association causal? Paediatric Respiratory Reviews, 22, 72–75. 27818068 10.1016/j.prrv.2016.08.010

[phy270622-bib-0012] Chavoshian, S. , Montazeri Ghahjaverestan, N. , Cao, X. , & Yadollahi, A. (2023). Modeling the effects of simulated obstructive apnea on elastic properties of lung in persons with asthma. D30 integrating osa and comorbidities for effective therapies: American Thoracic Society. A6514‐A.

[phy270622-bib-0013] Crisford, H. , Sapey, E. , Rogers, G. B. , Taylor, S. , Nagakumar, P. , Lokwani, R. , & Simpson, J. L. (2021). Neutrophils in asthma: the good, the bad and the bacteria. Thorax, 76(8), 835–844. 33632765 10.1136/thoraxjnl-2020-215986 PMC8311087

[phy270622-bib-0014] Davies, S. E. , Bishopp, A. , Wharton, S. , Turner, A. M. , & Mansur, A. H. (2019). The association between asthma and obstructive sleep apnea (OSA): a systematic review. The Journal of Asthma, 56(2), 118–129. 29641292 10.1080/02770903.2018.1444049

[phy270622-bib-0015] Eddy, R. L. , Svenningsen, S. , Kirby, M. , Knipping, D. , McCormack, D. G. , Licskai, C. , Nair, P. , & Parraga, G. (2020). Is computed tomography airway count related to asthma severity and airway structure and function? American Journal of Respiratory and Critical Care Medicine, 201(8), 923–933. 31895987 10.1164/rccm.201908-1552OC

[phy270622-bib-0016] Galbán, C. J. , Han, M. K. , Boes, J. L. , Chughtai, K. A. , Meyer, C. R. , Johnson, T. D. , Galbán, S. , Rehemtulla, A. , Kazerooni, E. A. , Martinez, F. J. , & Ross, B. D. (2012). Computed tomography–based biomarker provides unique signature for diagnosis of COPD phenotypes and disease progression. Nature Medicine, 18(11), 1711–1715. 10.1038/nm.2971 PMC3493851 23042237

[phy270622-bib-0017] Gottlieb, D. J. , & Punjabi, N. M. (2020). Diagnosis and management of obstructive sleep apnea: a review. Journal of the American Medical Association, 323(14), 1389–1400. 32286648 10.1001/jama.2020.3514

[phy270622-bib-0018] Guilleminault, C. , Stoohs, R. , Clerk, A. , Cetel, M. , & Maistros, P. (1993). The upper airway resistance syndrome. In Sleep apnea and Rhonchopathy (pp. 62–65). Karger Publishers.

[phy270622-bib-0019] Guo, F. , Svenningsen, S. , Kirby, M. , Capaldi, D. P. , Sheikh, K. , Fenster, A. , & Parraga, G. (2017). Thoracic CT‐MRI coregistration for regional pulmonary structure–function measurements of obstructive lung disease. Medical Physics, 44(5), 1718–1733. 28206676 10.1002/mp.12160

[phy270622-bib-0020] Gupta, S. , Siddiqui, S. , Haldar, P. , Entwisle, J. J. , Mawby, D. , Wardlaw, A. J. , Bradding, P. , Pavord, I. D. , Green, R. H. , & Brightling, C. E. (2010). Quantitative analysis of high‐resolution computed tomography scans in severe asthma subphenotypes. Thorax, 65(9), 775–781. 20805170 10.1136/thx.2010.136374 PMC2975950

[phy270622-bib-0021] He, Z. , Armoni Domany, K. , Nava‐Guerra, L. , Khoo, M. C. , DiFrancesco, M. , Xu, Y. , Khoo, M. C. K. , Mcconnell, K. , Hossain, M. M. , & Amin, R. (2019). Phenotype of ventilatory control in children with moderate to severe persistent asthma and obstructive sleep apnea. Sleep, 42(9), zsz130. 31175805 10.1093/sleep/zsz130

[phy270622-bib-0022] Iber, C. (2007). The AASM manual for the scoring of sleep and associated events: rules, terminology, and technical specification.

[phy270622-bib-0023] Jaimchariyatam, N. , Dweik, R. A. , Kaw, R. , & Aboussouan, L. S. (2013). Polysomnographic determinants of nocturnal hypercapnia in patients with sleep apnea. Journal of Clinical Sleep Medicine, 9(3), 209–215. 23493528 10.5664/jcsm.2480 PMC3578680

[phy270622-bib-0024] Kamio, Y. , Fujimura, M. , Futamata, H. , Matsubara, F. , & Matsuda, T. (1990). Potentiating effect of spirometric maneuver on bronchial responsiveness to methacholine in asthmatic subjects–The role of deep inspiration and forced expiration. Rinsho Byori the Japanese Journal of Clinical Pathology, 38(12), 1378–1382. 2082038

[phy270622-bib-0025] Kelly, V. J. , Brown, N. J. , Sands, S. A. , Borg, B. M. , King, G. G. , & Thompson, B. R. (2012). Effect of airway smooth muscle tone on airway distensibility measured by the forced oscillation technique in adults with asthma. Journal of Applied Physiology, 112(9), 1494–1503. 22362406 10.1152/japplphysiol.01259.2011

[phy270622-bib-0026] Kelly, V. J. , Sands, S. A. , Harris, R. S. , Venegas, J. G. , Brown, N. J. , Stuart‐Andrews, C. R. , King, G. G. , & Thompson, B. R. (2013). Respiratory system reactance is an independent determinant of asthma control. Journal of Applied Physiology, 115(9), 1360–1369. 23990243 10.1152/japplphysiol.00093.2013

[phy270622-bib-0027] Kittivoravitkul, P. , Kaw, R. , Hatipoğlu, U. , Wang, L. , & Aboussouan, L. S. (2016). Determinants of wake Pco2 and increases in wake Pco2 over time in patients with obstructive sleep apnea. Annals of the American Thoracic Society, 13(2), 259–264. 26636624 10.1513/AnnalsATS.201508-563OC

[phy270622-bib-0028] Kong, D.‐L. , Qin, Z. , Shen, H. , Jin, H.‐Y. , Wang, W. , & Wang, Z.‐F. (2017). Association of obstructive sleep apnea with asthma: a meta‐analysis. Scientific Reports, 7(1), 4088. 28642543 10.1038/s41598-017-04446-6 PMC5481327

[phy270622-bib-0029] Krishnan, R. , Canović, E. P. , Iordan, A. L. , Rajendran, K. , Manomohan, G. , Pirentis, A. P. , Smith, M. L. , Butler, J. P. , Fredberg, J. J. , & Stamenović, D. (2012). Fluidization, resolidification, and reorientation of the endothelial cell in response to slow tidal stretches. American Journal of Physiology‐Cell Physiology, 303(4), C368–C375. 22700796 10.1152/ajpcell.00074.2012 PMC3422985

[phy270622-bib-0030] Krishnan, R. , Park, C. Y. , Lin, Y.‐C. , Mead, J. , Jaspers, R. T. , Trepat, X. , Lenormand, G. , Tambe, D. , Smolensky, A. V. , Knoll, A. H. , Butler, J. P. , & Fredberg, J. J. (2009). Reinforcement versus fluidization in cytoskeletal mechanoresponsiveness. PLoS One, 4(5), e5486. 19424501 10.1371/journal.pone.0005486 PMC2675060

[phy270622-bib-0031] Lavoie, T. L. , Krishnan, R. , Siegel, H. R. , Maston, E. D. , Fredberg, J. J. , Solway, J. , & Dowell, M. L. (2012). Dilatation of the constricted human airway by tidal expansion of lung parenchyma. American Journal of Respiratory and Critical Care Medicine, 186(3), 225–232. 22679010 10.1164/rccm.201202-0368OC PMC3423451

[phy270622-bib-0032] Mat, D. Ö. , Firat, S. , Aksu, K. , Aksu, F. , & Duyar, S. Ş. (2021). Obstructive sleep apnea is a determinant of asthma control independent of smoking, reflux, and rhinitis. Allergy and Asthma Proceedings, 42(1), e25–e29. 33404398 10.2500/aap.2021.42.200098

[phy270622-bib-0033] Mauer, Y. , & Taliercio, R. M. (2020). Managing adult asthma: The 2019 GINA guidelines. Cleveland Clinic Journal of Medicine, 87(9), 569–575. 32868307 10.3949/ccjm.87a.19136

[phy270622-bib-0034] Montazeri Ghahjaverestan, N. , Chavoshian, S. , Cao, X. , Bradley, T. D. , Tarlo, S. M. , Stanbrook, M. , Chapman, K. R. , & Yadollahi, A. (2024). The effect of simulated obstructive apneas on mechanical characteristics of lower airways in individuals with asthma. Annals of Biomedical Engineering, 52(6), 1617–1624. 38433152 10.1007/s10439-024-03475-3

[phy270622-bib-0035] Pardo‐Manrique, V. , Ibarra‐Enríquez, C. D. , Serrano, C. D. , Sanabria, F. , & Fernandez‐Trujillo, L. (2024). Asthma and obstructive sleep apnea: Unveiling correlations and treatable traits for comprehensive care. Chronic Respiratory Disease, 21, 14799731241251827. 38717428 10.1177/14799731241251827 PMC11080759

[phy270622-bib-0036] Prasad, B. , Nyenhuis, S. M. , Imayama, I. , Siddiqi, A. , & Teodorescu, M. (2020). Asthma and obstructive sleep apnea overlap: what has the evidence taught us? American Journal of Respiratory and Critical Care Medicine, 201(11), 1345–1357. 31841642 10.1164/rccm.201810-1838TR PMC7258643

[phy270622-bib-0037] Prasad, B. , Nyenhuis, S. M. , & Weaver, T. E. (2014). Obstructive sleep apnea and asthma: associations and treatment implications. Sleep Medicine Reviews, 18(2), 165–171. 23890469 10.1016/j.smrv.2013.04.004

[phy270622-bib-0038] Quan, S. F. , Howard, B. V. , Iber, C. , Kiley, J. P. , Nieto, F. J. , O'Connor, G. T. , Rapoport, D. M. , Redline, S. , Robbins, J. , Samet, J. M. , & Wahl, P. W. (1997). The sleep heart health study: design, rationale, and methods. Sleep, 20(12), 1077–1085. 9493915

[phy270622-bib-0039] Saha, S. , Viswanathan, K. , Saha, A. , & Yadollahi, A. (2024). Feasibility of acoustic features of vowel sounds in estimating the upper airway cross sectional area during wakefulness: A pilot study. Speech Communication, 165, 103144.

[phy270622-bib-0040] Sakoe, H. , & Chiba, S. (1978). Dynamic programming algorithm optimization for spoken word recognition. IEEE Transactions on Acoustics, Speech, and Signal Processing, 26(1), 43–49.

[phy270622-bib-0041] Sánchez, T. , Castro‐Rodríguez, J. A. , & Brockmann, P. E. (2016). Sleep‐disordered breathing in children with asthma: a systematic review on the impact of treatment. Journal of Asthma and Allergy, 9, 83–91. 27143940 10.2147/JAA.S85624 PMC4844256

[phy270622-bib-0042] Sands, S. A. , Owens, R. L. , & Malhotra, A. (2016). New approaches to diagnosing sleep disordered breathing. Sleep Medicine Clinics, 11(2), 143–152. 27236052 10.1016/j.jsmc.2016.01.005 PMC5125379

[phy270622-bib-0043] Sato, S. , Saito, J. , Fukuhara, A. , Uematsu, M. , Suzuki, Y. , Rikimaru, M. , Kawamata, T. , Umeda, T. , Koizumi, T. , Togawa, R. , Sato, Y. , Nikaido, T. , Minemura, H. , Kanazawa, K. , Tanino, Y. , & Shibata, Y. (2021). Association between sleep characteristics and asthma control in middle‐aged and older adults: A prospective cohort study. Journal of Asthma and Allergy, 14, 325–334. 33854339 10.2147/JAA.S301444 PMC8040693

[phy270622-bib-0044] Saxena, D. , Imayama, I. , & Adrish, M. (2023). Revisiting asthma obstructive sleep apnea overlap: current knowledge and future needs. Journal of Clinical Medicine, 12(20), 6552. 37892689 10.3390/jcm12206552 PMC10607310

[phy270622-bib-0045] Sheel, A. W. , Guenette, J. A. , Yuan, R. , Holy, L. , Mayo, J. R. , McWilliams, A. M. , Lam, S. , & Coxson, H. O. (2009). Evidence for dysanapsis using computed tomographic imaging of the airways in older ex‐smokers. Journal of Applied Physiology (1985), 107(5), 1622–1628. 10.1152/japplphysiol.00562.2009 PMC2777797 19762522

[phy270622-bib-0046] Sorkness, R. , Teague, W. , Penugonda, M. , Fitzpatrick, A. , & National Institutes of Health NHL, Blood Institute's Severe Asthma Research P . (2011). Sex dependence of airflow limitation and air trapping in children with severe asthma. The Journal of Allergy and Clinical Immunology, 127(4), 1073–1074. 21310476 10.1016/j.jaci.2010.12.1079 PMC3078583

[phy270622-bib-0047] Svenningsen, S. , Kirby, M. , Starr, D. , Coxson, H. O. , Paterson, N. A. , McCormack, D. G. , Paterson, N. A. M. , & Parraga, G. (2013). What are ventilation defects in asthma? Thorax, 69, 203711. 10.1136/thoraxjnl-2013-203711 23956019

[phy270622-bib-0048] Taille, C. , Rouvel‐Tallec, A. , Stoica, M. , Danel, C. , Dehoux, M. , & Marin‐Esteban, V. (2016). Obstructive sleep apnoea modulates airway inflammation and remodelling in severe asthma. PLoS One, 11(3), e0150042. 26934051 10.1371/journal.pone.0150042 PMC4774979

[phy270622-bib-0049] Teodorescu, M. , Consens, F. B. , Bria, W. F. , Coffey, M. J. , McMorris, M. S. , Weatherwax, K. J. , Palmisano, J. , Senger, C. M. , Ye, Y. , Kalbfleisch, J. D. , & Chervin, R. D. (2009). Predictors of habitual snoring and obstructive sleep apnea risk in patients with asthma. Chest, 135(5), 1125–1132. 18849401 10.1378/chest.08-1273

[phy270622-bib-0050] Teodorescu, M. , Polomis, D. A. , Teodorescu, M. C. , Gangnon, R. E. , Peterson, A. G. , Consens, F. B. , Chervin, R. D. , & Jarjour, N. N. (2012). Association of obstructive sleep apnea risk or diagnosis with daytime asthma in adults. The Journal of Asthma, 49(6), 620–628. 22742082 10.3109/02770903.2012.689408 PMC3626099

[phy270622-bib-0051] Thomson, N. C. , Chaudhuri, R. , Spears, M. , Messow, C.‐M. , MacNee, W. , Connell, M. , Murchison, J. T. , Sproule, M. , & McSharry, C. (2015). Poor symptom control is associated with reduced CT scan segmental airway lumen area in smokers with asthma. Chest, 147(3), 735–744. 25356950 10.1378/chest.14-1119

[phy270622-bib-0052] Trepat, X. , Deng, L. , An, S. S. , Navajas, D. , Tschumperlin, D. J. , Gerthoffer, W. T. , Butler, J. P. , & Fredberg, J. J. (2007). Universal physical responses to stretch in the living cell. Nature, 447(7144), 592–595. 17538621 10.1038/nature05824 PMC2440511

[phy270622-bib-0053] Varatharajan, R. , Manogaran, G. , Priyan, M. K. , & Sundarasekar, R. (2018). Wearable sensor devices for early detection of Alzheimer disease using dynamic time warping algorithm. Cluster Computing, 21, 681–690.

[phy270622-bib-0054] Vasilescu, D. M. , Martinez, F. J. , Marchetti, N. , Galbán, C. J. , Hatt, C. , Meldrum, C. A. , Dass, C. , Tanabe, N. , Reddy, R. M. , Lagstein, A. , Ross, B. D. , Labaki, W. W. , Murray, S. , Meng, X. , Curtis, J. L. , Hackett, T. L. , Kazerooni, E. A. , Criner, G. J. , Hogg, J. C. , & Han, M. L. K. (2019). Noninvasive imaging biomarker identifies small airway damage in severe chronic obstructive pulmonary disease. American Journal of Respiratory and Critical Care Medicine, 200(5), 575–581. 30794432 10.1164/rccm.201811-2083OC PMC6727153

[phy270622-bib-0055] Wang, T.‐Y. , Lo, Y.‐L. , Lin, S.‐M. , Huang, C.‐D. , Chung, F.‐T. , & Lin, H.‐C. (2017). Obstructive sleep apnea accelerates FEV 1 decline in asthmatic patients. BMC Pulmonary Medicine, 17, 1–6. 28327130 10.1186/s12890-017-0398-2 PMC5361857

[phy270622-bib-0056] White, L. H. , & Bradley, T. D. (2013). Role of nocturnal rostral fluid shift in the pathogenesis of obstructive and central sleep apnoea. The Journal of Physiology, 591(5), 1179–1193. 23230237 10.1113/jphysiol.2012.245159 PMC3607865

[phy270622-bib-0057] Yadollahi, A. , Singh, B. , & Bradley, T. D. (2015). Investigating the dynamics of supine fluid redistribution within multiple body segments between men and women. Annals of Biomedical Engineering, 43, 2131–2142. 25632892 10.1007/s10439-015-1264-0

[phy270622-bib-0058] Zhang, G.‐Q. , Cui, L. , Mueller, R. , Tao, S. , Kim, M. , Rueschman, M. , Mariani, S. , Mobley, D. , & Redline, S. (2018). The national sleep research resource: towards a sleep data commons. Journal of the American Medical Informatics Association, 25(10), 1351–1358. 29860441 10.1093/jamia/ocy064 PMC6188513

